# Supplementary material for: Change in the magnetic configurations of tubular nanostructures by tuning dipolar interactions
Source: Sci Rep. 2018 Jul 6;8:10275. doi: 10.1038/s41598-018-28598-1 (PMC6035215; doi:10.1038/s41598-018-28598-1)
Supplement: Supplementary file 1 — Supplementary Information [file 41598_2018_28598_MOESM1_ESM.pdf]

Supplementary information for the article: Change in the magnetic configurations of tubular nanostructures by tuning dipolar interactions

H. D. Salinas,[1,*] J. Restrepo,[1,†] and Òscar Iglesias[2,‡]

[1]Grupo de Magnetismo y Simulación G+, Instituto de Física, Universidad de Antioquia. A.A. 1226, Medellín, Colombia

[2]Departament de Física de la Matèria Condensada and Institut de Nanociència i Nanotecnologia, Universitat de Barcelona, Av. Diagonal 647, 08028 Barcelona, Spain

I. CALCULATION OF EXCHANGE AND DIPOLAR ENERGIES

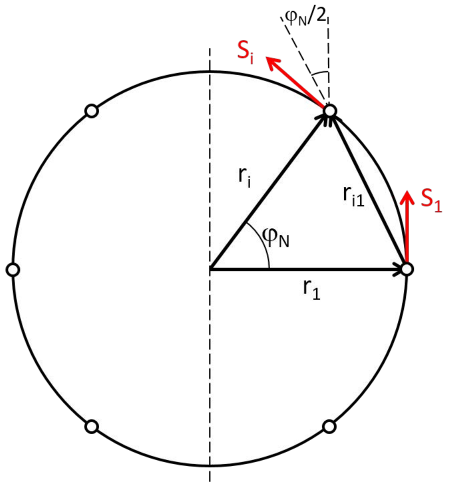

FIG. S1. Geometric parameters characterizing a nanotube with N spins per ring. Spin positions are shown by empty circles and directions tangent to the tube surface by red arrows. The angle between two consecutive spins separated by a distance r_{i1} is denoted by φ_N .

Let us consider a tube with spins pointing tangentially to the tube surface and aligned at an angle θ with respect to the tube axis. The components of any two consecutive spins can be written as (see Fig. S1)

$$\vec{S}_i = (0, \sin\theta, \cos\theta)$$
$$\vec{S}_{i+1} = (-\sin\theta\sin(\varphi_N/2), \sin\theta\cos((\varphi_N/2), \cos\theta) \ .$$

Then, the exchange energy is readily computed as all spins have z nn except for the N spins of the top and bottom layers which have only $z/2$ nn. Therefore, the exchange energy per spin and unit J is:

$$\epsilon_{ex}(\theta) = \frac{-z(NN_z - 2N) - z/2J(2N)}{NN_z}(\vec{S}_i \cdot \vec{S}_{i+1}) \quad (1)$$
$$= \frac{-zNN_z + 2zN - zN}{NN_z}(\vec{S}_i \cdot \vec{S}_{i+1}) \quad (2)$$
$$= \frac{-zNN_z + zN}{NN_z}(\vec{S}_i \cdot \vec{S}_{i+1}) \quad (3)$$
$$= -z\left(1 - \frac{1}{N_z}\right)\left[1 - \sin^2\theta(1 - \cos(\varphi_N/2))\right] \ . \quad (4)$$

For a (8,15) tube, this gives $\epsilon_{ex}(0) = -3.733$ and $\epsilon_{ex}(\pi/2) = -3.45$.

The dipolar energy can be also calculated exactly after a careful development by considering first the contribution of intralayer interactions and then the interlayer interactions between odd and even layers. Due to the cylindrical symmetry of the system, geometrical considerations allow to obtain compact expressions in J units. Thus, intralayer contribution is given by:

$$\epsilon_{dip,intra}(\theta) = \sum_{i=1}^{N/2} \frac{\omega_i}{d_i^3}(\cos\alpha_i - 3\cos^2\alpha_i'), \quad (5)$$

where i is an index labeling pairs of spins having a frequency to occur ω_i and

$$\cos\alpha_i = 1 - \sin^2\theta\,(1 - \cos(\varphi_i)) \quad (6)$$
$$\cos\alpha_i' = \sin\theta\cos(\varphi_i/2) \quad (7)$$
$$d_i = R\sqrt{2\,(1 - \cos(\varphi_i))} = 2R\sin(\varphi_1/2) \ . \quad (8)$$

This equation can be further transformed into a more compact formula if the sums are extended to N and angles α and α' are expressed as a function of θ, φ_i

$$\epsilon_{dip,intra}(\theta) = \sum_{i=1}^{N-1} \frac{1}{d_i^3}\left\{1 - \sin^2\theta\left[2 + \cos^2(\varphi_i/2)\right]\right\} \quad (9)$$
$$= \gamma\sum_{i=1}^{N-1} \frac{1}{d_i^3}\left\{1 - \frac{\sin^2\theta}{2}\left[5 + \cos\varphi_i\right]\right\} \ . \quad (10)$$

Odd interlayer contribution is given by:

$$\epsilon_{dip,odd}(\theta) = \sum_k \left(1 - \frac{(2k-1)}{N_z}\right)\sum_{i=1}^{N} \frac{(\cos\beta_i - 3\cos^2\beta_i')}{d_{i,2k-1}^3} \quad (11)$$

where k runs over layer indexes and

$$\cos\beta_i = 1 - \sin^2\theta\,(1 - \cos(\varphi_{2i-1}/2))$$
$$\cos\beta'_i = r_{ik,||}\sin\theta\cos(\varphi_{2i-1}/4)) + r_{ik,\perp}\cos\theta\ ,$$

where in turn

$$r_{ik,||} = \frac{d_{i,||}}{\sqrt{((2k-1)l)^2 + d_{i,||}^2}}$$
$$r_{ik,\perp} = \frac{(2k-1)l}{\sqrt{((2k-1)l)^2 + d_{i,||}^2}}$$
$$d_{i,||}^2 = 2R^2(1 - \cos((2i-1)\varphi_N/2)$$
$$d_{i,2k-1} = \sqrt{((2k-1)l)^2 + d_{i,||}^2}\ ,$$

being l the distance between two consecutive layers. Similarly, the even interlayer contribution is given by:

$$\epsilon_{dip,even}(\theta) = \sum_k \left(1 - \frac{2k}{N_z}\right)\sum_{i=1}^{N}\frac{(\cos\eta_i - 3\cos^2\eta'_i)}{d_{i,2k}^3} \quad (12)$$

where

$$\cos\eta_i = 1 - \sin^2\theta(1 - \cos\varphi_{i-1})$$
$$\cos\eta'_i = r_{ik,||}\sin\theta\cos(\varphi_{i-1}/2)) + r_{ik,\perp}\cos\theta$$

where in turn

$$r_{ik,||} = \frac{d'_{i,||}}{\sqrt{(2kl)^2 + d'^2_{i,||}}}$$
$$r_{ik,\perp} = \frac{2kl}{\sqrt{(2kl)^2 + d'^2_{i,||}}}$$
$$d'^2_{i,||} = 2R^2(1 - \cos\varphi_{i-1})$$
$$d_{i,2k} = \sqrt{(2kl)^2 + d'^2_{i,||}}\ .$$

For a (8,15) tube with $a = 0.5$, this expressions give $\epsilon_{dip}(0) = -13.105$ and $\epsilon_{dip}(\pi/2) = -17.289$.

II. ESTIMATION OF DIPOLAR INTERACTIONS

Let us estimate the order of magnitude of the dipolar interaction energy between magnetic dipoles that appears as a global factor D in our model Hamiltonian Eq. 1. For identical dipoles oriented parallel, it is given by $D = \frac{\mu_0}{4\pi}\frac{\mu^2}{d^3}$, where μ is the magnetization of a dipole and d the dipole-dipole separation. When the dipoles are described by individual atomic magnetic ions of a particular material, then inserting $\mu \sim n\mu_B$ and $d \sim xa$ ($a = 1\mathring{A}$), this gives $E_{dip} \sim 0.6\frac{n^2}{x^3}$ (K). For Co ($n = 1.7, x = 2.5$), we have $E_{dip} \sim 0.11$ K. Whereas for dipoles representing the total magnetization of a nanoparticle of volume V , the dipolar moment is $\mu = M_s V$. So that, for spherical particles of diameter D separated by a distance $d = \lambda D$, we have $E_{dip} = \frac{\mu_0}{4\pi}\frac{\pi}{6}M_S^2(\frac{D}{\lambda})^3$, and therefore, for typical $M_s = 5\times10^5$ A/m and $D = 10$ nm, we have $E_{dip} \sim 500$ K.

III. ADDITIONAL FIGURES

Spin configurations of the (8,15) for different values of γ corresponding to the profiles given in Fig. can also be visualized by using the Bloch sphere representation shown in Fig. S2, where all the 120 spins of the tube are drawn as points at the surface of a sphere according to their orientation in spherical coordinates (see Eq. 8). Configurations are colored according to their corresponding γ as shown in the legend of Fig. S2.

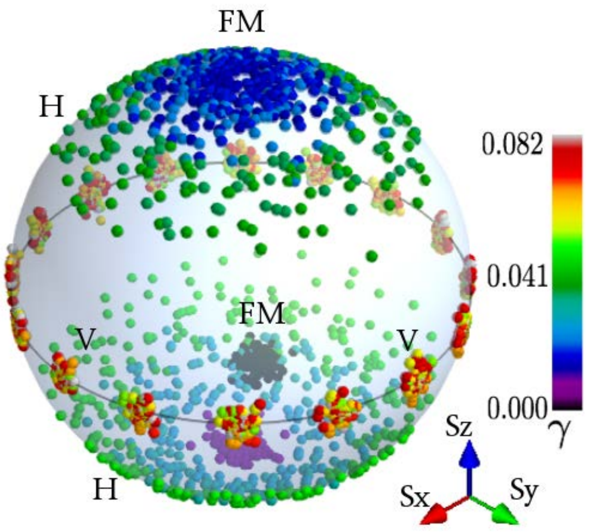

FIG. S2. Bloch sphere representation of the spin configurations shown in Fig. 8

Regarding the quasi-uniform states considered in the main text, we show in Fig. S3 the dependence of $\gamma^{\star}$ on the tube length for three tube radii. This is the value for which the energy of these states is independent of the angle θ.

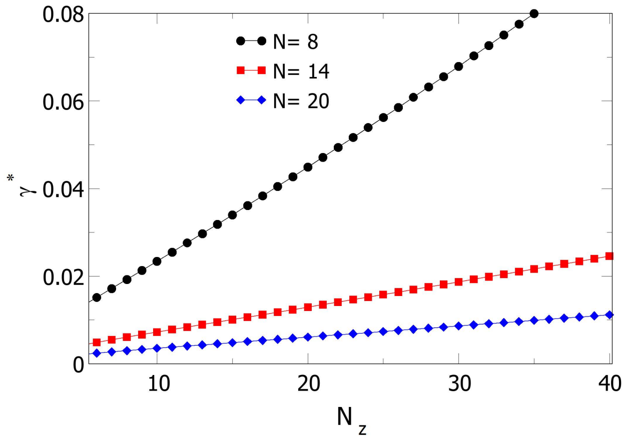

FIG. S3. Dependence of $\gamma^{\star}$ (the critical value for which magnetic configurations with spins tangent to the tube surface and having all the same angle θ have energy independent of θ) on the tube length N_z for tube radii indicated in the legend.

A representative magnetic configuration of the mixed states considered in the numerical calculations of the main text.

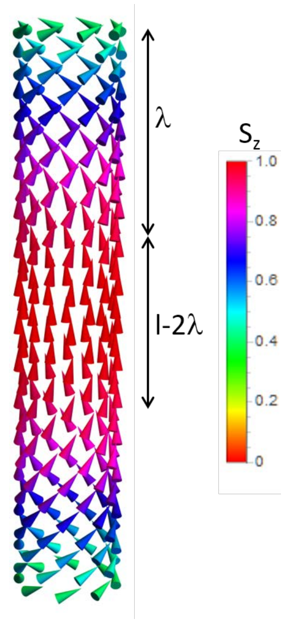

FIG. S4. Configuration of a (8,30) nanotube in the mixed state with $\theta_0 = 67^\circ$ and $\lambda = 14$.

Magnetic configurations obtained after MC simulated annealing for $\gamma = 0.05$ for tubes with the same dimensions as those presented in Fig. 10.

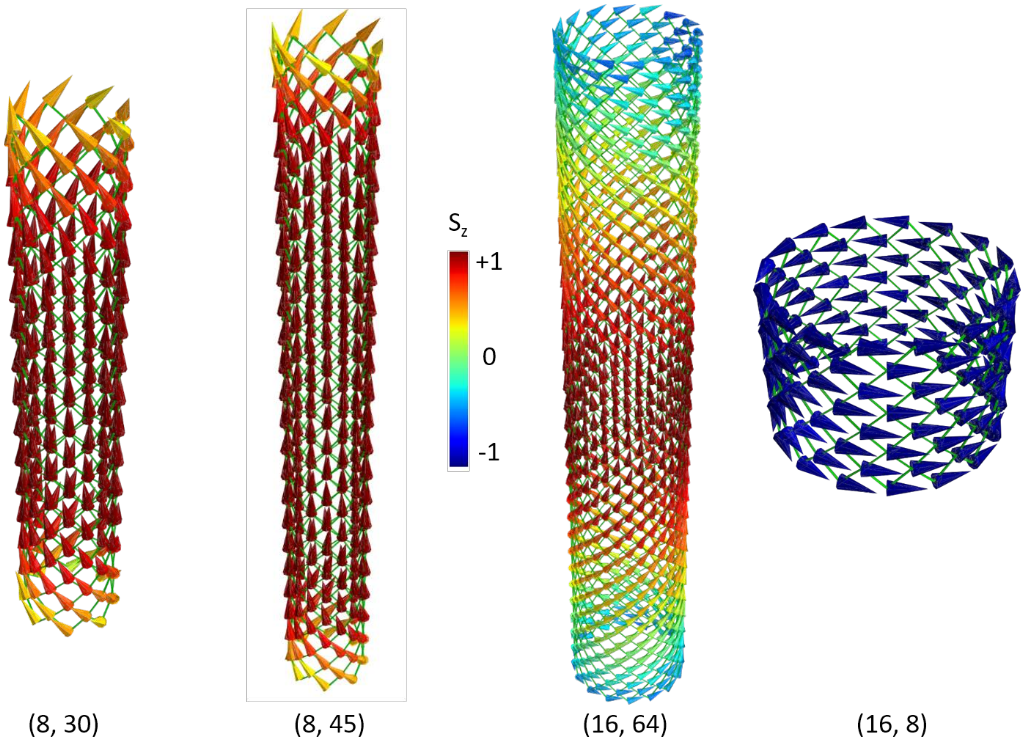

FIG. S5. Configurations obtained by MC simulation following simulated annealing process for $\gamma = 0.05$ and nanotubes with dimensions $(N, N_z) = (8, 30), (8, 45), (16, 64), (16, 8)$.

* hernan.salinas@udea.edu.co
† johans.restrepo@udea.edu.co
‡ oscar@ffn.ub.es; http://www.ffn.ub.es/oscar, http://nanomagn.blogspot.com
